# Potential Biological Roles of Exosomal Long Non-Coding RNAs in Gastrointestinal Cancer

**DOI:** 10.3389/fcell.2022.886191

**Published:** 2022-05-04

**Authors:** Fanhua Kang, Feng Jiang, Lingzi Ouyang, Shangjun Wu, Chencheng Fu, Ying Liu, Zhilan Li, Yu Tian, Xiaolan Cao, Xiaoping Wang, Qingchun He

**Affiliations:** ^1^ Department of Pathology, Xiangya Changde Hospital, Changde, China; ^2^ Department of Medicine, Xizang Minzu University, Xianyang, China; ^3^ Department of Emergency, Xiangya Hospital, Central South University, Changsha, China; ^4^ National Clinical Research Center for Geriatric Disorders, Xiangya Hospital, Central South University, Changsha, China; ^5^ Department of Emergency, Xiangya Changde Hospital, Changde, China

**Keywords:** gastrointestinal cancer, exosome, lncRNAs, pathogenesis, treatment

## Abstract

Exosomes, a type of extracellular vesicles (EVs), are secreted by almost all cells and contain many cellular constituents, such as nucleic acids, lipids, and metabolites. In addition, they play a crucial role in intercellular communication and have been proved to be involved in the development and treatment of gastrointestinal cancer. It has been confirmed that long non-coding RNAs (lncRNAs) exert a range of biological functions, such as cell metastasis, tumorigenesis, and therapeutic responses. This review mainly focused on the emerging roles and underlying molecular mechanisms of exosome-derived lncRNAs in gastrointestinal cancer in recent years. The biological roles of exosomal lncRNAs in the pathogenesis and therapeutic responses of gastrointestinal cancers were also investigated.

## Introduction

Gastrointestinal cancer has a high incidence worldwide (Dekker et al., 2019; Global Burden of Disease Cancer et al., 2019) and is mainly treated with surgery and chemotherapy (Ajani et al., 2016; Benson et al., 2018; Benson et al., 2020). Due to the inconspicuous early clinical symptoms, gastrointestinal cancer is usually diagnosed at an advanced stage, resulting in high recurrence and mortality rates. Exosomes can transfer long non-coding RNAs (lncRNAs) to recipient cells, suggesting that they can affect biological functions such as regulating the occurrence and progression of gastrointestinal cancer (Li et al., 2019a; Kalluri and LeBleu, 2020). The studies of exosome-derived lncRNAs can help us to further elucidate the underlying molecular mechanisms of cancer progression and provide potential biomarkers for early diagnosis and targeted therapies for gastrointestinal cancer patients.

### Gastrointestinal Cancer

Gastric cancer (GC) is the fifth most common cancer and the third most deadly cancer worldwide (Smyth et al., 2020). Colorectal cancer (CRC) is the fourth leading cause of cancer death in the world (Deng et al., 2021; Lu et al., 2021). Gastrointestinal cancer is usually treated with surgery, chemotherapy, targeted therapies, and so on. Patients receiving early diagnosis and treatment for gastrointestinal cancer have better prognosis than those diagnosed at an advanced stage (Luo and Li, 2019). Existing diagnostic methods almost exclusively rely on invasive procedures such as digestive endoscopy and pathological biopsy, which are difficult to be widely used for screening. Therefore, it is of great significance to investigate new biomarkers for early diagnosis and targeted therapies.

### Exosomes

**FIGURE 1:**
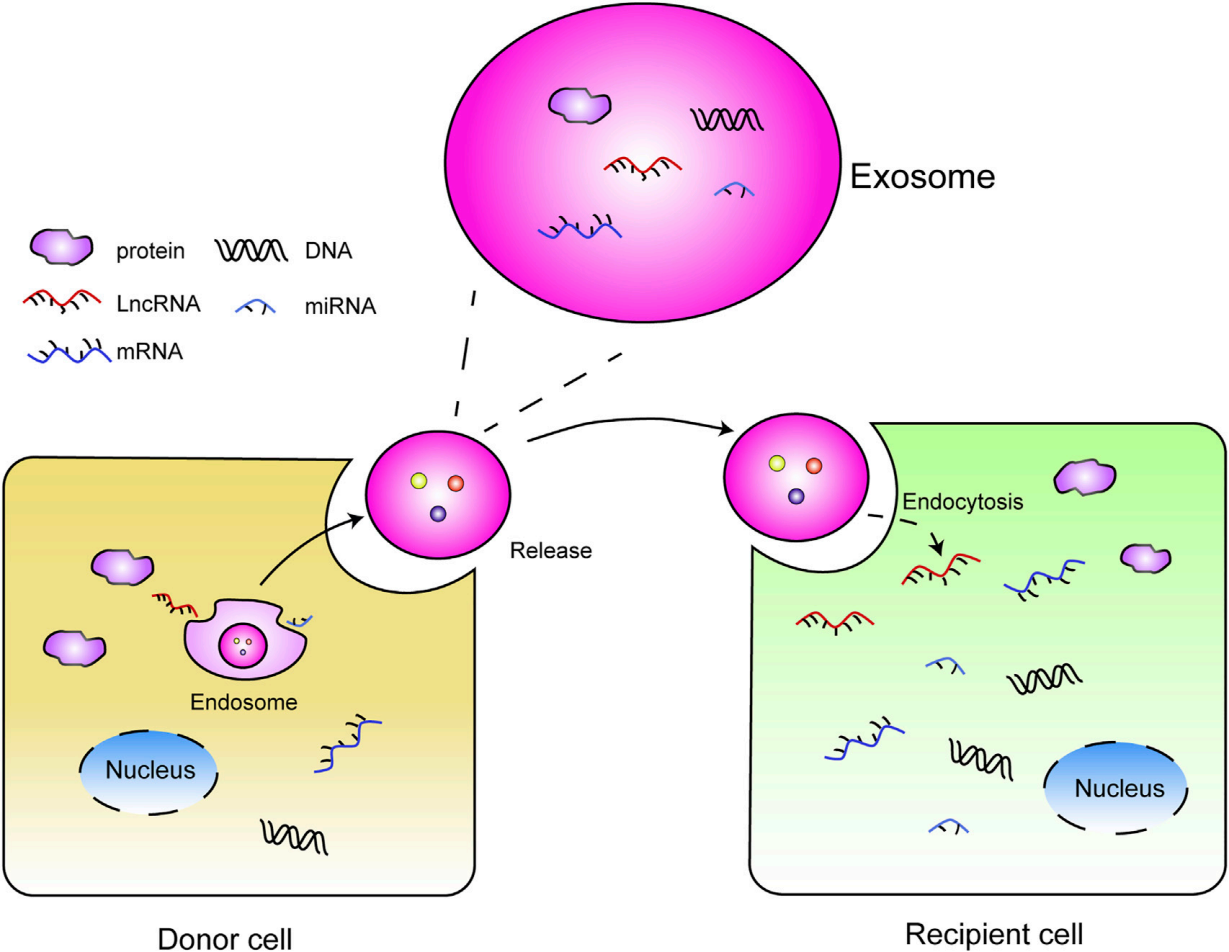
Intercellular communication: donor cells release the exosomes with contents (DNAs, RNAs, protein) that are received by recipient cells *via* endocytosis, and the cargoes contained in exosome exert function in recipient cells.

Exosomes are small, single-membrane, secreted organelles that contain selected proteins, nucleic acids, lipids, glycoconjugates metabolites, and so on (Kumar et al., 2019; Thakur et al., 2021; Thakur et al., 2020; Qiu et al., 2019), ranging from 50 to 150 nm in diameter (∼100 nM, medially) (Phan et al., 2018; Thakur et al., 2022). Exosomes play essential roles in intercellular communication. Additionally, exosome-associated nucleic acids, proteins, and metabolites can alter the functional consequence in recipient cells through autocrine and paracrine signaling (Figure 1), thus participating in the cancer progression and treatment (Zhang and Yu, 2019).

### lncRNAs

The majority of expressed transcripts do not encode proteins, and the transcripts (>200 nt) in length are broadly classified as lncRNAs, once dismissed as “junk” RNA (Yan et al., 2017; Yan et al., 2019; Kong et al., 2021; Wei et al., 2021; Yue et al., 2021). Nowadays, the latest studies have demonstrated that lncRNAs can modulate gene expression by mediating translational inhibition or functioning as competitive endogenous RNAs (ceRNAs) (Iyer et al., 2015; Jacob et al., 2017; Richtig et al., 2017; Rinn and Chang, 2020). It has been confirmed that lncRNAs play critical roles in cancer progression and metastasis (Wang et al., 2021a; Ghafouri-Fard et al., 2021). Recently, exosomal lncRNAs have been reported to regulate multiple biological processes of cancers, such as apoptosis, proliferation, migration, and angiogenesis (Sun et al., 2018; Li et al., 2019b; Chen et al., 2019; Cheng et al., 2020; Kok and Yu, 2020; Behera et al., 2021). For instance, exosomal lncRNA LNMAT2 can promote lymphangiogenesis and lymph node metastasis in bladder cancers (Chen et al., 2019). In another study, exosomal FMR1-AS1 can activate TLR7-NFκB signaling pathway to induce and promote the progression of esophageal squamous cell carcinoma (Li et al., 2019b). These exosomal-derived lncRNAs have been proved to be potential biomarkers for the diagnosis and prognosis of various cancers, including gastrointestinal cancers (Wang et al., 2018; Guo et al., 2020).

## Biological Roles of Exosomal lncRNAs in Gastrointestinal Cancer

LncRNAs have been proved to be significantly correlated with the occurrence and progression of gastrointestinal neoplasms. Exosomes are small-walled nanovesicles secreted by a variety of cells and involved in the progression of gastrointestinal cancer, such as proliferation, metastasis, and drug resistance by transferring lncRNAs (Table 1). Moreover, emerging evidence has showed the underlying molecular mechanisms of the exosomal lncRNAs in the biological processes of malignant cells.

**TABLE 1:** The function of exosomal lncRNAs in gastrointestinal cancer.

LncRNA	Type of Cancer	Biological Function	Refs
H19	CRC	Tumorigenesis and proliferation	38
UCA1	CRC	Proliferation; promotes cetuximab resistance	39,55
HEIH	GC	Tumorigenesis	40
LINC01559	GC	Proliferation	41
ZFAS1	GC	Proliferation	42
FRLnc1	GC	Proliferation	43
SNHG10	CRC	Proliferation	45
KCNQ1OT1	CRC	Proliferation	46
CRNDE-h	*CRC*	Lymph node metastasis	49
RPPH1	CRC	Metastasis	50
APC1	CRC	Angiogenesis and metastasis	52
MALAT1	CRC	Metastasis	53
PCGEM1	GC	Invasion and metastasis	54
HOTTIP	GC	Promoting cisplatin resistance	56
CCAL	CRC	Promoting oxaliplatin resistance	57
CRNDE	GC	Promoting cisplatin resistance	58

### Roles of Exosomal lncRNAs in Cell Proliferation

The abundantly expressed lncRNA H19 has been found in many human cancers. In CRC, the expression level of H19 of carcinoma-associated fibroblasts (CAFs) was significantly higher than that of normal fibroblasts (NFs). Ren and his colleague found that the CAF-derived exosomes can transfer lncRNA H19 to neighboring cells and activate the Wnt/β-catenin signaling pathway in CRC cells, thus facilitating the tumorigenesis and cell proliferation (Ren et al., 2018). In both tissue and plasma exosomes of CRC patients, the expression level of lncRNA-UCA1 increases. Mechanistically, UCA1 can regulate the expression of MYO6 *via* miRNA-143 sponge as a ceRNA. Researchers observed that after treatment with exosomal UCA1 from CRC patients, the expression of miR-143 decreases but MYO6 expression increases, thus promoting CRC cell proliferation (Luan et al., 2020). lnc HEIH can be encapsulated by exosomes and then transferred into natural gastric cells to stimulate the expression of EZH2, causing high methylation of the GSDME promoter and promotion of tumorigenesis (Lu et al., 2020). LINC01559 can enhance the proliferation, migration, and stemness characteristics of GC cells. Wang et al. found that the expression of LINC01559 is upregulated in mesenchymal stem cells (MSCs) compared with that in GC cells, and then exosomes from MSCs can transfer LINC01559 into GC cells to promote the cell progression by activating the PI3K/AKT signaling pathway (Wang et al., 2020). lncRNA ZFAS1 was proved to be involved in cell cycle regulation. The exosomes can promote GC cell proliferation through the transfer of ZFAS1 (Pan et al., 2017). Zhang et al. found that GC cells treated with the exosomes containing FOXM1-related lncRNA (FRLnc1) can enhance GC cell proliferation and migration. The FRLnc1 knockdown in GC cells can induce cell cycle arrest and cell apoptosis (Zhang et al., 2021). In addition, some studies have shown that the exosomal lncRNAs derived from cancer cells can regulate the cancer immune microenvironment, such as immunosuppressants and immune escape, to promote cancer progression. Wang et al. observed that lncRNA RP11-323N12.5 secreted by GC cells is associated with Treg cell–induced immunosuppression. RP11-323N12.5 can be transferred into T cells by exosome delivery and then enhance YAP1 transcription in T cells, thus leading to promotion of GC cells (Wang et al., 2021b). Exosomal lncRNA SNHG10 derived from CRC cells can regulate NK cell function by upregulating INHBC expression. It can significantly downregulate the release of perforin-1 and granzyme B to inhibit NK cell growth and then promote CRC cell growth (Huang et al., 2021). Xian’s group found that lncRNA KCNQ1OT1 derived from CRC cells can promote CRC progression. Mechanistically, exosomes can transfer KCNQ1OT1 via autocrine to mediate the miR-30a-5p/USP22 pathway, then regulate the ubiquitination of PD-L1 and inhibit CD8^+^ T-cell responses (Xian et al., 2021). Taken together, these results suggested that dysregulated exosomal lncRNAs may be meaningful biomarkers for cancer cell proliferation.

### Roles of Exosomal lncRNAs in Cell Metastasis

It has been reported that exosomal lncRNA CRNDE-h levels are significantly correlated with lymph node metastasis and distant metastasis in the CRC (Liu et al., 2016). Early studies have demonstrated that the level of T-helper 17 cells is closely related to regional lymph node metastasis in CRC (Lee et al., 2017). CRC-derived exosomes can promote Th17 cell differentiation by transmitting CRNDE-h, and then promote lymph node metastasis (Sun et al., 2021). lncRNA RPPH1 can bind to TUBB3 to prevent its ubiquitination and degradation and induce EMT and cell metastasis of CRC cells. In addition, RPPH1 can mediate macrophage M2 polarization by being transferred to exosomes-bearing macrophages to promote CRC cell metastasis (Liang et al., 2019). In another study, macrophage M2 polarization was observed in BRAFV600E mutation of CRC, resulting in more angiogenesis and lymphangiogenesis in the microenvironment. Zhi et al. believed that this phenomenon may be related to the abundance of some lncRNAs in exosomes (Zhi et al., 2021). As a vital mediator of APC, lncRNA-APC1 directly regulates the stability of Rab5b mRNA, hence reducing the exosome secretion of CRC cells. Exosomes derived from lncRNA-APC1–silenced CRC cells can activate the MAPK pathway and then enhance actin refactoring and angiogenesis, thereby accelerating cell metastasis (Wang et al., 2019a). Exosomal lncRNA MALAT1 can function as a ceRNA via miR-26a/26b sponge and then enhance phosphorylation in PI3K/Akt/mTOR pathway and FUT4-associated fucosylation, involved in CRC cell metastasis (Xu et al., 2020a). Piao et al. found that the PCGEM1 expression was dramatically higher in hypoxia-cultured GC cells (HGC) than in normoxic-cultured cells (NGC). Moreover, PCGEM1 can be transferred from HGC cells to NGC cells by being packaged into exosomes, enhancing invasion and metastasis of NGC cells (Piao et al., 2021). Therefore, investigating the roles of exosomal lncRNA in the cellular metastasis can provide a promising strategy for targeted anti-metastatic therapies in gastrointestinal cancer.

### Roles of Exosomal lncRNAs in Cell Chemoresistance

In addition to regulating cell proliferation, exosomal lncRNA UCA1 also mediates chemoresistance in CRC. UCA1 expression in cetuximab-resistant cells is significantly higher than that in cetuximab-sensitive cells. Further studies showed that recipient cells can obtain greater cetuximab resistance via exosomal transmission of UCA1 from cetuximab-resistant CRC cells (Yang et al., 2018). Exosomal lncRNA HOTTIP promotes cisplatin resistance by activating HMGA1. Mechanistically, exosomal HOTTIP can sponge miR-218 to mediate HMGA1 expression (Wang et al., 2019b). lncRNA CCAL can reduce the sensitivity of oxaliplatin (Oxa) and 5-FU, and CAF-derived exosomes can transfer CCAL to CRC cells, thus promoting Oxa resistance of CRC cells (Deng et al., 2020). In *in vitro* experiments, the expression level of lncRNA CRNDE was overexpressed in M2-polarized macrophage-derived exosomes (M2-exo) and it was encapsulated into exosomes to be transferred from M2 macrophages to GC cells. Studies showed that after GC cells are treated with M2-exo of silenced CRNDE, their cisplatin sensitivity was significantly enhanced (Xin et al., 2021). Collectively, exosomal lncRNA may help clarify the underlying molecular mechanisms of therapeutic resistance in gastrointestinal cancer and provide promising therapeutic strategies.

## Clinical Application of Exosomal lncRNAs in Gastrointestinal Cancer

Exosomal lncRNAs from serum, plasma, and other body fluids are stable due to the particularity of their molecular structures, serving as ideal biomarkers and therapeutic targets for gastrointestinal cancer patients (Table 2).

**TABLE 2:** The clinical application of exosomal lncRNAs in gastrointestinal cancer.

lncRNA	Type of Cancer	Expression	Type of Biomarker	Reference
lncRNA-GC1	GC	High expression	Early diagnosis and prognosis	36
LNCV6_116109, LNCV6_98390, LNCV6_38772, LNCV_108266, LNCV6_84003, LNCV6_98602	CRC	High expression	Diagnosis	59
HOTTIP	CRC, GC	Low/mediate expression	Diagnosis and prognosis	60,61
ADAMTS9-AS1	CRC	Low expression	Diagnosis	62
lnc-GNAQ-6:1	GC	Low expression	Diagnosis	63
lncUEGC1	GC	High expression	Diagnosis	64
PCSK2-2:1	GC	Low expression	Diagnosis	65
CEBPA-AS1	GC	High expression	Diagnosis	66
MIAT	GC	High expression	Diagnosis	67
SLC2A12-10:1	GC	High expression	Diagnosis and prognosis	68
H19	GC	High expression	Diagnosis	69
LINC00659	CRC	Low expression	therapeutic target	70
GAS5	CRC	Low expression	therapeutic target	71

### Exosomal lncRNAs as Diagnostic and Prognostic Biomarkers

Hu’s group found that a group of six exosomal lncRNAs (LNCV_108266, LNCV6_84003, LNCV6_116109, LNCV6_98390, LNCV6_38772, and LNCV6_98602) are significantly overexpressed in the plasma of CRC patients, and they may serve as a promising non-invasion biomarker for diagnosis of CRC (Hu et al., 2018). The low/mediate expression of exosomal-derived lncRNA HOTTIP has been found to be significantly associated with poor overall survival. Oehme et al. found that patients with low/mediate expression of HOTTIP in primary CRC tissue may have a poor prognosis (Oehme et al., 2019). It was also found that with the increase in expression levels of exosomal HOPPIT, the depth of tumor invasion and TNM stages also increased in GC patients, indicating that exosomal HOPPIT may serve as a potential biomarker for the diagnosis and prognosis of GC (Zhao et al., 2018). The expression of serum exosomal lncRNA ADAMTS9-AS1 in CRC patients is significantly downregulated compared with that in healthy controls, suggesting that the exosomal ADAMTS9-AS1 may be a novel biomarker for the diagnosis of CRC (Li et al., 2020a). In GC patients, the expression of serum exosomal lnc-GNAQ-6:1 is reduced, but more studies are needed to determine whether it can be used as a new diagnostic biomarker for GC (Li et al., 2020b). In stage I GC patients, plasma exosomal lncRNA lncUEGC1 exhibits high diagnostic value compared with plasma exosomal lncUEGC2 and serum CEA, which may serve as a primary diagnostic biomarker for GC (Lin et al., 2018). The expression of the serum exosomal lncRNA PCSK2-2:1 is significantly downregulated in GC patients compared with that in healthy controls and is associated with tumor size, tumor stage, and venous invasion, suggesting that exosomal RNA PCSK2-2:1 may be a new prospective biomarker for GC diagnosis (Cai et al., 2019). Guo and his colleagues found that the expression levels of exosomal lncRNA-GC1 are closely associated with tumor burden, and they considerably accelerate from early to advanced stages with the progression of GC, showing that the expression levels of serum exosomal lncRNA-GC1 can serve as a potential early diagnostic biomarker and monitor the progression of GC (Guo et al., 2020). In the study of Piao’s group, the ROC curve and AUC value of plasma exosomal lncRNA CEBPA-AS1 are significantly higher than those of traditional markers with better sensitivity and specificity, suggesting that CEBPA-AS1 may be used as a novel diagnostic biomarker for GC (Piao et al., 2020). Xu et al. found that the high expression levels of serum exosomal lncRNA MIAT were significantly correlated with differentiation, lymphatic metastasis, and TNM stages of GC patients. In addition, in the serum of treated GC patients, the expression levels of exosomal MIAT were significantly reduced, indicating that the serum exosomal lncRNA MIAT may be a potential biomarker for monitoring GC progression (Xu et al., 2020b). In recent studies, the expression levels of exosomal lncRNA SLC2A12-10:1 were found to be dramatically associated with size, differentiation, TNM stages, and lymph node metastasis of GC tumors. The aberrantly expressed exosomal SLC2A12-10:1 may have a great potential to be a new biomarker for cancer diagnosis and prognosis (Zheng et al., 2020). Zhou and his colleagues found that the AUC curve of exosomal lncRNA H19 is much higher than that of any other traditional biomarker in GC, which may serve as an appropriate diagnostic marker for GC (Zhou et al., 2020). In general, exosomal lncRNAs show enormous potential to become ideal biomarkers for diagnosis and prognosis of gastrointestinal cancers.

### Exosomal lncRNAs as Therapeutic Targets

CAF-derived exosomal lncRNA LINC00659 can downregulate miR-342-3p and increase ANXA2 expression, which accelerates EMT and the progression of CRC cells (Zhou et al., 2021), and it may be targeted as a novel strategy for CRC treatment. Liu et al. found that the expression of lncRNA GAS5 in CRC patients is significantly downregulated, but miR-221 increases both in tissue, plasma exosomes, suggesting that the overexpression of lncRNA GAS5 may restrain the expression of miR22 (Liu et al., 2018). It deserves further study whether cancer growth can be inhibited by exosome-transferred GAS5. The research into the mechanism of exosomal lncRNAs in gastrointestinal cancer progression may have great significance for targeted therapies.

## Conclusion

The burden of gastrointestinal cancer is increasing worldwide. How to make an early diagnosis of gastrointestinal cancers and provide early treatment for them is a great challenge. Finding novel treatment methods or biomarkers may be a prospective strategy. Exosomes play a vital role in intercellular communication by releasing a wide variety of biological molecules, such as miRNAs, lncRNAs, proteins, and their complexes (Thakur et al., 2022). In recent years, the studies of lncRNAs have shown that lncRNAs play a crucial role in occurrence and progression of cancers. Since the structure of exosomes can protect lncRNAs from degradation, exosomal lncRNAs display great potential to become emerging non-invasion biomarkers for cancer diagnosis, prognosis, and treatment. Recent studies have also showed that exosomal lncRNAs have better sensitivity and specificity than traditional markers. However, the detailed mechanism and biological functions of most exosomal lncRNAs remain unclear. In addition, we found that some exosomal lncRNAs are similarly expressed in different cancers, which will bring challenges to the clinical application of exosomal lncRNAs. It may become a significant research direction to find more specific exosomal lncRNAs and further study their underlying molecular mechanisms, aiming to assist in diagnosis and serve as targets for targeted therapies.
